# Illumina and Nanopore methods for whole genome sequencing of hepatitis B virus (HBV)

**DOI:** 10.1038/s41598-019-43524-9

**Published:** 2019-05-08

**Authors:** Anna L. McNaughton, Hannah E. Roberts, David Bonsall, Mariateresa de Cesare, Jolynne Mokaya, Sheila F. Lumley, Tanya Golubchik, Paolo Piazza, Jacqueline B. Martin, Catherine de Lara, Anthony Brown, M. Azim Ansari, Rory Bowden, Eleanor Barnes, Philippa C. Matthews

**Affiliations:** 1 Nuffield Department of Medicine, Medawar Building, University of Oxford, South Parks Road, Oxford, OX1 3SY UK; 2 Wellcome Centre for Human Genetics, Roosevelt Drive, Oxford, OX3 7BN UK; 3 Department of Infectious Diseases and Microbiology, Oxford University Hospitals NHS Foundation Trust, John Radcliffe Hospital, Headley Way, Oxford, OX3 9DU UK; 4 Big Data Institute, Old Road, Oxford, OX3 7FZ UK; 5 Imperial BRC Genomics Facility, Imperial College, London, UK; 6 Gastroenterology and Hepatology Clinical Trials Facility, Oxford University Hospitals NHS Foundation Trust, John Radcliffe Hospital, Oxford, OX3 9DU UK; 7 Department of Hepatology, Oxford University Hospitals NHS Foundation Trust, John Radcliffe Hospital, Oxford, OX3 9DU UK; 8 NIHR Oxford Biomedical Research Centre, Oxford University Hospitals NHS Foundation Trust, John Radcliffe Hospital, Oxford, OX3 9DU UK

**Keywords:** Hepatitis B virus, Genomics, Next-generation sequencing, Virology

## Abstract

Advancing interventions to tackle the huge global burden of hepatitis B virus (HBV) infection depends on improved insights into virus epidemiology, transmission, within-host diversity, drug resistance and pathogenesis, all of which can be advanced through the large-scale generation of full-length virus genome data. Here we describe advances to a protocol that exploits the circular HBV genome structure, using isothermal rolling-circle amplification to enrich HBV DNA, generating concatemeric amplicons containing multiple successive copies of the same genome. We show that this product is suitable for Nanopore sequencing as single reads, as well as for generating short-read Illumina sequences. Nanopore reads can be used to implement a straightforward method for error correction that reduces the per-read error rate, by comparing multiple genome copies combined into a single concatemer and by analysing reads generated from plus and minus strands. With this approach, we can achieve an improved consensus sequencing accuracy of 99.7% and resolve intra-sample sequence variants to form whole-genome haplotypes. Thus while Illumina sequencing may still be the most accurate way to capture within-sample diversity, Nanopore data can contribute to an understanding of linkage between polymorphisms within individual virions. The combination of isothermal amplification and Nanopore sequencing also offers appealing potential to develop point-of-care tests for HBV, and for other viruses.

## Introduction

Chronic hepatitis B virus (HBV) infection affects an estimated 250–290 million individuals worldwide, resulting in around 800,000 deaths from chronic liver disease and hepatocellular carcinoma each year^1,2^. The status of HBV infection as a globally important public health problem is highlighted by United Nations Sustainable Development Goals, which set a target for HBV elimination by the year 2030^3^. An improved understanding of the molecular biology, epidemiology, infection dynamics and pathophysiology of HBV is a crucial step towards reducing the global burden of HBV disease. Despite the availability of a robust prophylactic vaccine and safe suppressive antiviral therapy, HBV has remained endemic - and neglected - in many populations^4^. Large-scale virus genome sequencing to provide more complete genetic information at the population and individual level can shed light on the limitations of current interventions^5^, and inform new strategies for elimination. New sequencing initiatives are required with improved methodologies that are efficient, accurate, sensitive and cost-effective^6^.

In the context of clinical and public health settings, HBV sequencing can provide information that is useful in characterizing virus genotype, potential transmission networks, drug and vaccine resistance, and aspects of the dynamics of infection^5,7,8^. Traditional Sanger sequencing can derive consensus sequences (usually of sub-genomic fragments), and next-generation technologies such as Illumina can interrogate within-sample diversity at the whole-genome level. Sequencing complete virus genomes at depth, while also preserving mutation-linkage information (ie. complete haplotypes), remains an important goal. Such data will inform more accurate phylogenetic characterisation of viral quasispecies within infected hosts, which can in turn be interpreted to study virus transmission and the evolutionary dynamics of drug and immune escape^6^.

‘Third generation’ (i.e. single-molecule) sequencing approaches including those based on nanopores (Oxford Nanopore Technologies, ONT)^9,10^, have the potential to revolutionise virus genome sequencing by producing genome-length reads that encompass all of the mutations within a single virus particle. In addition, Nanopore technology is portable and provides sequence data in real time, potentially enabling sequencing as a point-of-care test. However, Nanopore sequencing has been adopted with caution because of its high raw error rates^11^. While error-corrected Nanopore consensus sequences may be sufficiently accurate for many uses, raw-read accuracy remains a concern if it is to be used for the assessment of within-sample (between-molecule) diversity. One strategy to reduce error rates from single source molecules is to create concatemeric (chain-like) successive copies of each template, so that a single concatemer contains several reads of each base from the original molecule. This approach has been demonstrated in the circularization of 16 S bacterial DNA sequences followed by ‘rolling circle amplification’ (RCA) using a high-fidelity DNA polymerase^12^.

**Figure 1:**
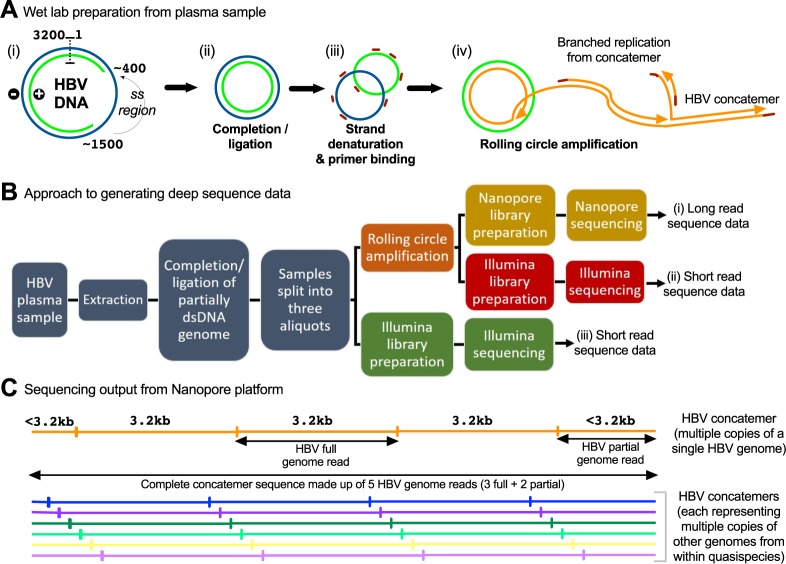
Schematic diagrams to show the pipeline for HBV sample processing. (**A**) (i) HBV genomes comprise partially double-stranded DNA in human plasma samples; (ii) completion-ligation (CL) derives a completely double-stranded DNA molecule; (iii) the complete dsDNA molecule is denatured and primers (red) bind; (iv) rolling circle amplification (RCA) generates genome concatemers, containing multiple end-to-end copies of the HBV genome (shown in orange). Amplification may also arise de novo due to priming along the length of the concatemer, creating a branched structure (primers shown in red). (**B**) Flow diagram to illustrate sample processing from from plasma through to HBV genome sequencing on Nanopore (yellow) and Illumina (red and green) platforms. This work flow allowed us to undertake a comparison between data derived from Illumina sequencing with RCA vs. without RCA, and comparison of RCA followed by sequencing using Illumina vs. Nanopore. Comparison of Nanopore with RCA vs. without RCA was not possible due to the requirement for amplification of HBV DNA prior to Nanopore sequencing (as shown in Table 2). (**C**) The sequence dataset derived from Nanopore comprises concatemeric reads comprising multiple reads of the same HBV genome (shown in orange). As indicated, concatemers containing three full length genomes also contain first and last segments that are partial (<3.2 kb). Other HBV genomes from among the quasispecies are represented by other individual concatemers (shown in blue, green, purple).

HBV has an unusual, circular, partially double-stranded (ds) DNA genome of approximately 3.2 kB (Fig. 1A(i))^6^. The combination of double- and single-stranded DNA in a single molecule can cause technical problems for sequencing, since library preparation methods are usually specific for either double- or single-stranded DNA templates. HBV isolates have previously been sequenced with Nanopore technology using full-length and sub-genomic PCR approaches to enrich for HBV sequences^13,14^. Whilst these approaches worked well in the studies when applied to high viral load samples, in both publications correction was only possible at the consensus level, with one study having a raw read error rate of ~12%^13^, and the other unable to definitively confirm putative minority variants detected in the minION reads^14^. In this study we build on a published method for HBV enrichment and amplification from plasma^15,16^, which generates intermediates that are suitable for sequencing by Nanopore or Illumina. We implement novel analytical methods to exploit concatemeric reads in improving the accuracy of Nanopore sequencing of HBV for use in research and clinical applications.

## Results

### Completion ligation and rolling circle amplification prior to illumina sequencing of full-length HBV genomes

We applied sequencing methods (as shown in Fig. 1) to plasma from three different adults with chronic HBV infection (Table 1). We first set out to convert the partially dsDNA viral genome (Fig. 1A(i)) to a complete dsDNA HBV molecule using a completion-ligation (CL) method (Fig. 1A(ii))^16^, so that sequencing libraries could be generated using kits that require dsDNA as input. Following CL, genomes were amplified by the use of primers (Fig. 1A(iii) and rolling circle amplification (RCA; Fig. 1A(iv))^15,16^. We confirmed an increase in HBV DNA after RCA by comparing extracted DNA to RCA products using qPCR (Suppl Methods 1). Using DNA products derived from from CL followed by RCA (Fig. 1B(ii)) and from CL alone without an RCA step (Fig. 1B(iii)), we prepared sequencing libraries and sequenced them using an Illumina MiSeq instrument.

**Table 1:** Details of samples used for HBV sequencing.

Sample Name	1331 (HEP-1361)	1332 (HEP-1317)	1348 (HEP-1407)	1331/2 mix	Plasmid^17^
HBV DNA viral load (log_10_ IU/ml)^a^	>8.23	>8.23	>8.23	n/a	n/a
HBeAg status	+	+	+	n/a	n/a
HBV genotype^b^	C	E	C	C/E	D
**Nanopore sequencing results**					
Flowcell chemistry	R9.4	R9.4	R9.5.1	R9.5.1	R9.4
Total reads	293,178	1,449,744	2,892,475	844,602	225,601
Total bases	1.01 Gb	4.28 Gb	5.97 Gb	2.30 Gb	1.05 Gb
Pass reads	257,321	1,296,131	1,674,661	510,966	148,892
Pass, trimmed bases	0.96 Gb	4.09 Gb	3.77 Gb	1.59 Gb	0.77 Gb
HBV reads	3,201	17,281	10,628	13,153	132,557
Proportion of HBV reads^c^	1.2%	1.3%	0.6%	2.6%	89%
Complete concatemer sequences^d^ generated	208	795	32	671	297
Error rate following consensus correction^e^	0.88%	0.92%	1.20%	n/a	n/a
Error rate following k-mer-error correction^e^	0.29%	0.28%	0.32%	n/a	n/a
**Illumina sequencing results**					
lLaboratory approach	CL; RCA	CL; RCA	CL; RCA	CL; RCA	
Total reads	2,786,410; 759,454	1,710,996; 1,049,818	3,070,746; 3,044,962	2,988,466; 3,056,144	n/a
Read length (bp)	255; 255	255; 255	300; 300	300; 300	n/a
High quality reads	2,772,002; 756,356	1,705,744; 1,043,028	3,044,686; 3,013,046	2,952,414; 3,024,164	n/a
HBV reads (mapping to all genotypes)	21,669; 18,078	3,531; 23,816	29,935; 39,430	60,140; 152,498	n/a
Proportion of HBV reads	0.79%; 2.39%	0.21%; 2.28%	0.98%; 1.31%	2.04%; 5.04%	n/a
HBV reads mapping to genotype reference, deduplicated	12,712; 12,990	2,502; 16,780	13,966; 23,430	24,202; 71,264	n/a

Patients were adults with chronic HBV infection, enrolled through a cohort in Oxford, UK.

HBV = hepatitis B virus, HBeAg = hepatitis B e-antigen, n/a = not applicable, CL = completion ligation, RCA = rolling circle amplification. Note that the yields and pass rates of these runs varied. They were conducted on different flowcells and a different basecaller version was used. As it stands, this assay is not quantitative, and we anticipate that yields of high-quality HBV reads will continue to vary as the Nanopore technology develops. ^a^Upper limit of quantification for HBV DNA viral load is 8.23 log_10_. ^b^Genotypes determined by read mapping to genotype consensus sequences derived from HBVdb sequences. ^c^The proportion of reads mapped to HBV out of the total number of pass reads ^d^Defined as concatemers with ≥3 full genome reads, with all genome reads mapping to either the plus or minus strand (see methods). ^e^Based on comparison with Illumina sequence and calculated at sites with <1% variation in Illumina data.

**Figure 2:**
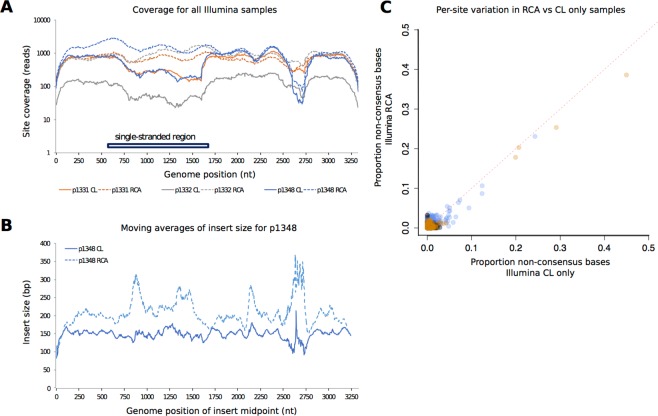
Comparison between HBV sequence coverage and diversity in Illumina sequences generated by completion/ligation (CL) alone, versus CL followed by Phi 29 rolling circle amplification (RCA). (**A**) Read depth across the length of the HBV genome for samples 1331, 1332 and 1348 by CL alone (solid lines) and by CL + RCA (dashed lines); (**B**) Average insert size across the HBV genome for sample 1348; (**C**) Variation detected in sequences based on CL alone, vs. CL + RCA. Each point represents a genome position with read depth >100. For each of these positions, variation is measured as the proportion of non-consensus base calls, and plotted for both sample types. The red dotted line indicates y = x. In all plots points are coloured by patient as follows: 1331 = orange, 1332 = grey, 1348 = blue.

Both the CL and CL + RCA methods generated Illumina sequencing data that covered the whole HBV genome for all three samples (Fig. 2A). The relative drop in coverage across the single-stranded region of the HBV genome disappeared after RCA, suggesting a preferential amplification of intact whole HBV genomes.

We observed a region of reduced coverage, corresponding approximately to nt 2500–2700, in all samples (Fig. 2A). Further examination of the sample with the sharpest drop in coverage across this region (sample 1348) revealed a drop in the density of insert ends in the region (Suppl Fig. 1) and resulting disruption to insert size (Fig. 2B), consistent with inefficient digestion by the Nextera transposase. Reasons for the reduced coverage are unclear; no nicks in the HBV genome have been described in this region, but there may be some secondary structure present. GC content may also be a contributing factor: GC bases in the region nt 2500–2700 account for 35–37.5% in the Illumina consensus sequences, in contrast to the rest of the genome, where GC content is 48–49.5%.

To investigate the possible effects of RCA on the representation of within-sample diversity, we compared variant frequencies between CL and CL + RCA. Only 2% of sites had variants at a frequency >0.01 and there appeared to be a consistent reduction in estimated frequency in RCA compared with CL alone (Fig. 2C), but overall this effect appears to be very minor for the samples we have studied.

### Completion ligation and rolling circle amplification facilitates nanopore sequencing of full-length HBV genomes

**Figure 3:**
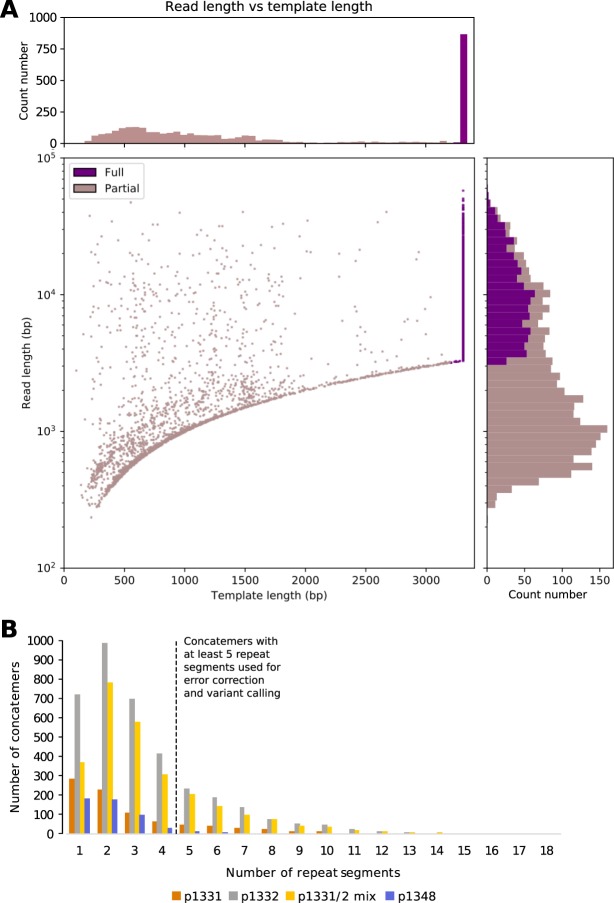
HBV sequence data generated by Nanopore sequencing following completion/ligation (CL) of the genome and rolling circle amplification (RCA). (**A**) Read length and template length of all reads generated from sample 1331. ‘Template length’ refers to the length of the primary alignment of the read, based on a concatenated reference genome. Template length is capped at 3.3 kb. Reads with alignments ≥3.2 kb in length are considered ‘full length’ concatemers; these are shown in dark purple. (**B**) Plot to show the number of repeat segments in ‘full length’ concatemers. This is equal to the number of segments that a read is chopped into based on the repeated location of an anchor sequence (see methods for details). Reads with ≥5 repeat segments will contain ≥3 full length copies of the HBV genome, as shown in Fig. 1C. These are taken forward for error correction and further analysis.

We used the material generated by RCA for Nanopore sequencing on the MinION (ONT) (Fig. 1B(i)). Reads mapping to HBV accounted for 0.6–1.3% of all sequences derived from individual patient samples (Table 1). The majority of the remainder of reads mapped to the human genome (Suppl Fig. 2). The reads included concatemers of the full-length HBV genome (as illustrated in Fig. 1C) reaching up to 16 HBV genomes per concatemer sequence, with a median of 1–2 HBV genomes (Fig. 3A,B). The number of reads passing quality criteria required for downstream analysis (described in the methods section) are shown in Table 1.

### RCA sequencing followed by nanopore does not produce chimeric sequences

In order to ascertain whether recombination occurred between different viral genomes during RCA or Nanopore sequencing^12^, we sequenced a mixture of two plasma samples (1331 and 1332, genotypes C and E respectively), producing 3,795 HBV reads (of any length) with a primary mapping to genotype C and 9,358 HBV reads with a primary mapping to genotype E. Of these, 148 genotype C and 532 genotype E reads were in the form of complete concatemer sequences (defined as containing ≥3 full HBV genomes) and between them they contained 4,805 HBV full or partial genome reads (for definitions, see Fig. 1C). We scored the similarity of each HBV genome read to the 1331 and 1332 Illumina consensus sequences at each of 335 sites that differed between the two consensus sequences, classifying genome segments as genotype C or genotype E if they matched the respective consensus at ≥80% of sites (Suppl Fig. 3). No complete concatemer sequences contained a mixture of geno-C and geno-E HBV genome reads. Only 6/4,805 HBV genome reads (either full or partial length) could not be classified in this way, each of which constituted either a partial genome covering <8 marker sites, or a low-quality sequence matching variants from both genotypes (Suppl Fig. 3). Thus, we found no evidence that the RCA process generates recombined sequences.

### Error correction in nanopore data

Among all Nanopore complete concatemer sequences with ≥3 full genome reads (as defined in Fig. 1C), 11.5% of positions differed from the Illumina consensus sequence for that sample. Given Nanopore raw error rates and the observation that the Illumina data contained very few within-host variants, we considered that the majority of such differences were likely to be Nanopore sequencing errors. Correcting such errors would allow us to phase true variants into within-sample haplotypes, improving on the information available from Illumina sequencing alone.

As a first step in correcting Nanopore sequencing errors at the level of the complete concatemer sequence, we took the consensus of all HBV genome reads (both full and partial reads) in each concatemer. Such an approach involves a trade-off between increasing the minimum number of HBV genome reads per concatemer for inclusion to optimise error correction, versus increasing the number of complete concatemer sequences under consideration to maximise sensitivity for assessment of within-sample diversity.

To assess error rates, we compared corrected Nanopore sequences with the Illumina consensus, considering only those sites with <1% variation in the Illumina data. For sample 1331, analysis of all sequences containing ≥3 HBV full genome reads maximised the total number of distinct complete concatemer sequences available for analysis (n = 208), and resulted in 0.88% of positions with a consensus call different from Illumina. Changing the criteria to be more stringent, we analysed only concatemers containing ≥8 HBV full genome reads, giving us a smaller pool of concatemer sequences (n = 41) but reducing the mean proportion of sites that varied from the Illumina consensus to 0.51% (Suppl Table 1).

In order to reduce the error rate, while maximising the number of complete concatemer sequences, we adopted a refined error correction method based on two assumptions:

(i) Basecaller errors are randomly distributed across all complete concatemer sequences, whereas true genetic variants are consistently seen in HBV genome reads within a subset of concatemers;
(ii) Systematic sequencing errors tend to be associated with a particular sequence context, or k-mer (Suppl Fig. 4A). In many cases, the error rate associated with a particular k-mer differs from that associated with its reverse complement (with the exception of longer homopolymers). Thus, basecaller errors often appear to be strand-specific, whereas true genetic variants can be seen with equal probability in forward and reverse strand reads (Suppl Figs 4B and 5). Note that the RCA process is such that forward reads may have had either strand of the original circular HBV genomes as their original template, and similarly for reverse reads (Fig. 1A).

**Figure 4:**
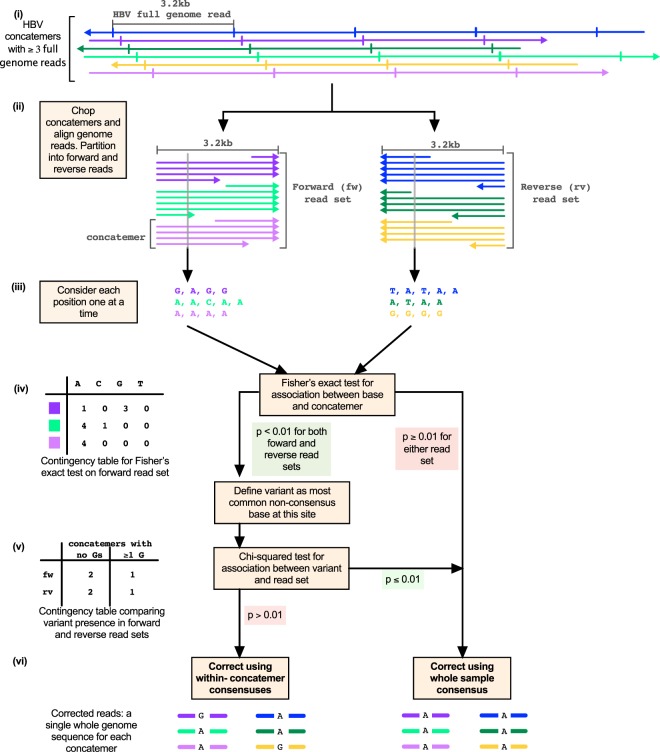
Error correction in Nanopore HBV sequence dataset. Schematic to depict the identification and removal of basecaller errors. (i) 6 concatemers containing at least three full length HBV reads (plus two partial genome reads) are illustrated. The same 6 colours are used throughout this figure to indicate the concatemer of origin. (ii) Concatemers are shown chopped into full and partial genome reads, partitioned according to whether they align to the forward (LHS) or reverse (RHS) strand of the reference. (iii) Each position is considered independently. Aligned bases for the position in question are collected and grouped by concatemer, as shown by the coloured list of bases. (iv) Fisher’s Exact test is conducted to determine the strength of association between base and concatemer within each read set. In the example contingency table on the left for the forward read set, guanine is found consistently in the dark purple concatemer but not in the other two concatemers. (v) The example contingency table illustrates conducting a Chi-squared test to see whether concatemers containing the variant, guanine, are significantly more common in one of the two read sets (forward or reverse). Significance criteria for the tests in (iv) and (v) are shown on the flow diagram, with significant results highlighted in green and non-significant results highlighted in red. (vi) The corrected concatemer sequence for this position of interest is illustrated, for the case where concatemers are corrected using the whole sample consensus base (right), and for the case where concatemers are corrected using the within-concatemer consensus base (left). Note that the p-values from step (iv) are also used to assign a quality score to each variant, as described in the methods and reported in Suppl Table 3.

To identify sites of true genetic polymorphism, for the data generated from each sample we tested for an association between base and concatemer at each site, to determine whether some bases were consistently found in particular concatemers at any one site, as described in assumption (i) above. For this we analysed forward and reverse strand reads separately, requiring that an association was found in both read sets (forward and reverse) for the site to be considered truly polymorphic (Fig. 4(ii–iv)).

We additionally tested each site for an association between variant (presence/absence within a concatemer) and strand (forward/reverse), thus sites where the potential variant showed significant strand bias were not considered truly polymorphic (Fig. 4(v)). We corrected polymorphic sites using the within-concatemer consensus base, whereas sites that failed this test were corrected using the whole-sample consensus base for all concatemers (Fig. 4(vi)). The result was a single, corrected, HBV genome haplotype for each original complete concatemer sequence. Further details on this error correction procedure are provided in the methods.

**Figure 5:**
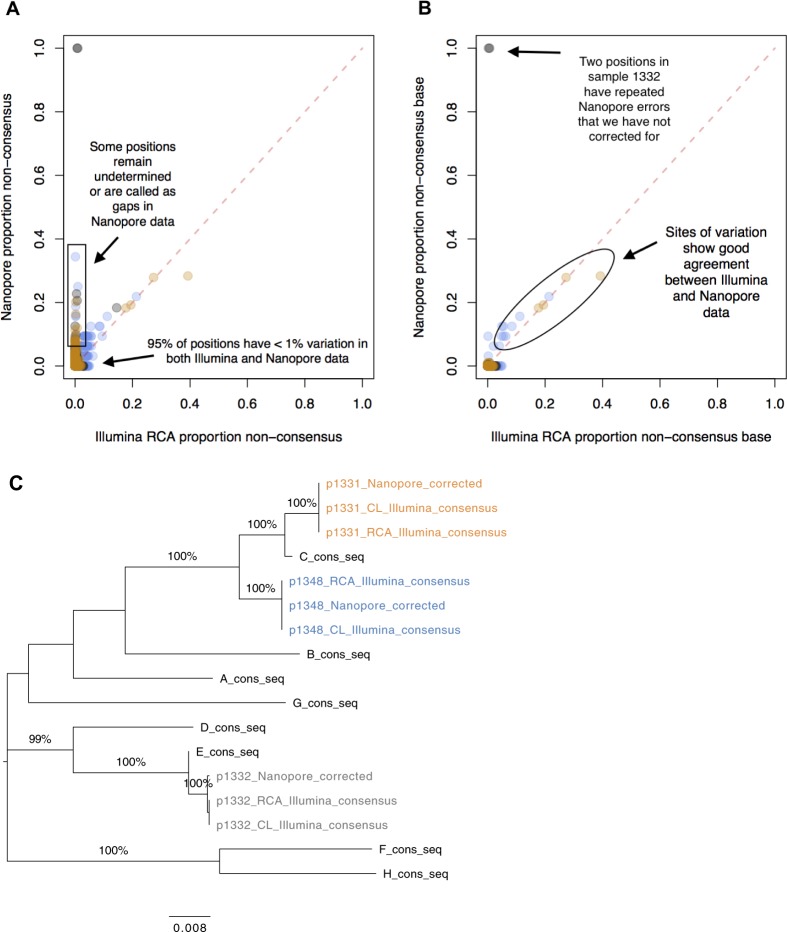
Comparison of HBV sequence data generated by Nanopore vs Illumina platforms, using completion/ligation (CL) and rolling circle amplification (RCA). (**A**) Proportion of non-consensus calls at each position in the genome based on Nanopore (y-axis) vs Illumina (x-axis), for samples 1331 (orange), 1332 (grey) and 1348 (blue). Note that the ‘proportion of non-consensus calls’ represents a slightly different quantity in the two data sets: in the Illumina data, an individual concatemer may give rise to multiple reads covering a position, where as in the Nanopore data each concatemer results in only one base call. The two sites with 100% variation in Nanopore data are positions 1741–1742 in sample 1332. These lie adjacent to a homopolymer repeat and the high error rate is the result of misalignment when the homopolymer length is miscalled. Positions that are only ever called as ambiguous in the Nanopore data are omitted from this plot (totalling 5 in both 1331 and 1348). Otherwise, sites called as ambiguous (‘N’) or gaps (‘−’) are considered ‘non-consensus’. (**B**) As for panel A, but sites called as ambiguous or gaps are not considered ‘non-consensus’ any more; only alternate bases (A,C,G,T) are included in the ‘non-consensus’ total. (**C**) Phylogenetic tree of consensus sequences for samples 1331 (orange), 1332 (grey) and 1348 (blue) generated by Illumina following CL, Illumina following CL + RCA, and Nanopore following CL + RCA sequencing, together with reference sequences for Genotypes A-H. Bootstrap values ≥80% are indicated. Scale bar shows substitutions per site.

The final corrected Nanopore sequences differed from the Illumina-derived consensus at an average of <0.4% of sites for the three samples studied (Table 1). We noted that many of these differences were called as gaps (‘−’) or ambiguous sites (‘N’) in the Nanopore data, so the proportion of sites which had been called as an incorrect base was even lower (Fig. 5).

### Detection of true genetic variants in nanopore data

We then switched our attention to the sites which our Nanopore correction method had highlighted as genuine variants. All variants with >10% frequency in the Illumina RCA data were also detected by the Nanopore method, and frequencies from the two methods showed good concordance (Fig. 5A,B). When considering those variants that appeared at >10% frequency in corrected Nanopore concatemers, all were confirmed as genuine by their presence in the Illumina data (Suppl Table 3). Hence, the Nanopore approach shows good sensitivity and specificity for calling mid-low frequency variants.

We also used the set of complete concatemer sequences to derive a within-patient consensus sequence from the Nanopore data. For two out of three samples (1331 and 1348) we found this to be identical to the final consensus sequences for Illumina using CL +/− RCA (excluding 5 sites in each sample which were called as ‘N’s in the Nanopore consensus) (Fig. 5C). In the third case (1332), the Nanopore consensus differed at just two sites, located next to a homopolymer (GGGGG).

**Figure 6:**
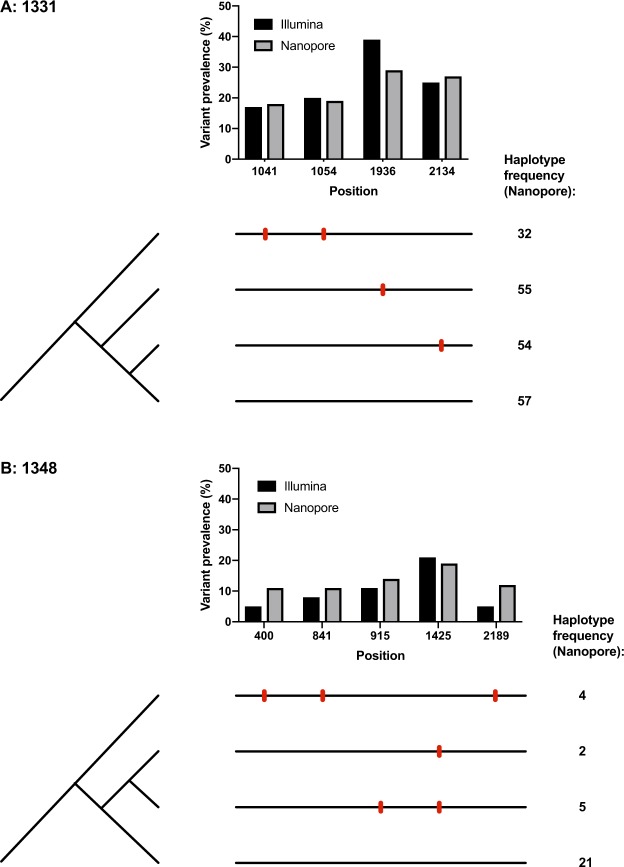
Maximum parsimony trees showing haplotypes called using corrected Nanopore concatemers. For each of samples 1331 and 1348, the high quality variant calls (as listed in Suppl Table 3) were used as a definitive set of variant sites. For each corrected concatemer, the haplotype was called according to the corrected bases at these variant sites. Haplotypes that occurred at >1% frequency within the sample are shown here, with the additional exclusion of one haplotype in sample 1331 that occurred at much lower frequency than those shown (only 3 occurrences) and did not allow for construction of a maximum parsimony tree without homoplasy. Counts of haplotypes are recorded on the left hand side, while the frequency of the variants in the Illumina and Nanopore data is indicated in bar charts along the top of each diagram. Variants (bases differing from the consensus) are indicated with a red bar on the horizontal lines that represent the whole-genome haplotypes. A potential method for assigning quality scores to haplotype calls, based on the length and number of the concatemers supporting the call, is presented in Suppl Methods 3. Based on these calculations, all haplotypes with ≥ 3 concatemers supporting them have a phred-based quality score of >30.

A primary advantage that Nanopore (long-read data) offers over Illumina (short-read data) is the ability to generate full-length haplotypes, providing insights into the epistatic interactions between polymorphisms at different loci. This is illustrated by quantifying the proportion of genomes derived from Nanopore data that represent a specific haplotype, characterised by combinations of multiple polymorphisms (Fig. 6). For example, we were able to identify linkage between two mutations in sample 1348, spaced 1,789 bp apart in 4/32 whole genome haplotypes (at sites nt 400 and nt 2189, Suppl Table 3). Comparing this to Illumina data, the same polymorphisms are detected at similar frequencies but cannot be assigned to a single haplotype in combination. Thus, accurate haplotyping with Nanopore facilitates improved insight into within-host population structure.

### Sequence data generated from a plasmid by nanopore sequencing

To further evaluate our methods, we applied our RCA amplification, library preparation, Nanopore sequencing and variant detection pipeline to an HBV plasmid^17^. No genetic variants were detected within this sample, as anticipated for clonal genetic material. The corrected consensus sequence differed from the published plasmid sequence^17^ at only 1/6820 positions (excluding 26 sites which were called as ‘N’s). This difference was the result of a homopolymer miscall, similar to the case in 1332. These results confirm the high fidelity of the RCA enrichment step and the accuracy of our bioinformatic approach for sequence data generated by Nanopore.

### Sequence availability

Consensus sequences for our Illumina completion-ligation (MK720628, MK720629, MK720632), Illumina RCA (MK720627, MK720630, MK720631) and Nanopore sequences (MK321264, MK321265, MK321266) have been deposited into Genbank. HBV reads generated from the sequencing platforms have been made available via the European Nucleotide Archive with the study accession number PRJEB31886.

## Discussion

Robust generation of full-length HBV sequence data is an important aspiration for improving approaches to clinical diagnosis (including point-of-care diagnostics and detection of co-infections), patient-stratified management, molecular epidemiology, and long-term development of cure strategies, following precedents set by work in HIV^18^. However, the unusual biology of the HBV genome has represented a significant challenge for whole-genome sequencing to date^6^.

We here demonstrate and compare the use of two different sequencing platforms to generate full length HBV sequences from clinical samples. Illumina deep sequencing approaches allow determination of diversity and detection of minor variants, but have the disadvantage of short reads that do not permit the reconstruction of complete viral haplotypes. In contrast, our new Nanopore protocol may under-estimate the total diversity present within a sample, but allows us to gain confidence in the generation of whole HBV genome haplotypes. Existing approaches can already determine mixed or highly-diverse infections^18,19^ however, additional insight into the linkage between polymorphisms, and developing methods to track divergent quasispecies, may yield important benefits in understanding the evolutionary biology and clinical outcomes of HBV infection. A comparison of the pros and cons of different sequencing approaches is summarised in Table 2.

**Table 2:** Comparison of three methods of deriving HBV sequence data.

	Sanger	Illumina	Nanopore
Laboratory time (sample prep)	3 h	3 h	3 h
Laboratory time (generation of sequences)	1–6 h	20–56 h	48 h
Amount of DNA required for input	30–75 ng	1–50 ng	0.1–1 µg
Sensitivity (derivation of sequences from low viral load samples)	High	Medium	Low
Genome coverage	100% (in individual fragments)	100% (with predictable areas of lower coverage)	100%
Depth of sequencing (representation of quasispecies)	Represents consensus only; biased according to selection of primers	Reliable detection of quasispecies, but may require enrichment steps which can produce bias	Currently limited by need for enrichment, which can introduce bias
Likely error rate	0.1% per read	0.2–0.7% per read^35,36^	~12% per read^37^, 0.3% per concatemer (after correction)
Long read vs short read	Short (estimated 600–1000 base pairs per read)^38^	Short (estimated 150–600 base pairs per read)^38^	Long (only limited by size of library loaded onto flowcell)
Key attributes	Rapid, cheap generation of consensus sequence	Representation of complete diversity present within a sample	Generation of full length viral haplotypes; portable test offers potential for point of care diagnosis
Key concerns	No representation of diversity; bias dependent on selection of primers; need for amplification in fragments	Potential errors in reconstruction of complete viral haplotypes. Expensive set up; requires lab infrastructure	May under-represent diversity; poor sensitivity in the absence of an amplification step

Many users of Nanopore technology are primarily interested in obtaining an accurate full-length consensus sequence for diagnostic purposes. Error correction tools such as Nanopolish^20^ are sufficient for such applications, but methodological adjustments are required for the analysis of intra-host diversity. Our analysis highlights that, aside from homopolymer errors, many errors in raw Nanopore sequence data are k-mer-specific. The approach used in this study, using both genome-length concatemers and strand specificity to distinguish k-mer-specific errors from genuine diversity, facilitates error correction at the per-read level. The approach did not introduce any unexpected diversity when applied to a ‘clonal’ population of plasmid HBV genomes, adding to our confidence that the polymorphisms we detect in the final corrected dataset reflect genuine genetic variants rather than Nanopore sequencing errors.

For a given number of genomes in a concatemer, there is a trade-off between the amount of data available for analysis, relative to the potential for accurate error correction (Suppl Table 1). Thus, using three genomes in a concatemer produces the largest data-set but a relatively higher error rate, while increasing the threshold to six genomes per concatemer reduces the available data-set for analysis, but also lowers the error rate. The approach taken by any individual study might therefore alter the threshold for the minimum number of concatenated genomes, according to the question being asked (a study seeking to quantify maximum possible diversity would benefit from analysing a smaller number of genomes per concatemer, while a study requiring highly robust error correction might raise the threshold for genome copy numbers in each concatemer). Future optimisation focused on increasing the number of long concatemers will improve the specificity and sensitivity of variant identification and thereby the resolution of low-frequency variants on haplotypes. Long concatemers also improve the confidence with which low frequency haplotypes can be called and linkage established (Suppl Methods 3 and Suppl Fig. 9).

As a new technology, Nanopore sequencing is currently still evolving rapidly, with updates to basecalling algorithms, kits and the flowcell chemistry being frequently released. Our bioinformatic methods are based on general principles of the technology, and hence have shown applicability across samples sequenced using different flowcell and basecaller versions (Table 1). At present, this assay is not quantitative, and in this study we observed considerable variability in total yields and proportion of mapped HBV reads between Nanopore sequencing runs. However, it is reasonable to expect that the generation of high quality HBV data will increase as further updates improve total yields and raw accuracy rates.

In chronic HBV infection, the hepatitis B e-antigen (HBeAg)-positive phase of infection is frequently characterised by high viral loads and low viral diversity, as in the samples described here. It has been hypothesised that reduced immune-mediated selection during the HBeAg phase of infection is allowing the unconstrained replication of conserved viral populations^21,22^, explaining the low diversity we observed in our samples. Marked increases in viral diversity have been described prior to and immediately after HBeAg seroconversion, coinciding with reductions in viral load^22^. Samples from the seroconversion phase are relatively unusual in clinical practice, and focused studies undertaken within large, diverse clinical cohorts will be needed to identify and study individuals in this stage of chronic infection. Further work with larger numbers of samples, including different disease context and phenotypes (e.g. acute infection, transmission networks, patients with a wide range of viral loads, HBeAg-negative status, chronic disease including cancer and cirrhosis), will be of interest in characterising the utility of these different methods for diversity analyses, including identification of specific sequence polymorphisms and determination of within and between host diversity. Optimisation for lower viral loads is particularly important for the approach to become widely applicable. Broadly speaking, sensitivity can be optimised through viral enrichment (for example using probe-based selection^19,23^ and/or by using laboratory approaches that deplete human reads^24.^

Our results demonstrate that our approach is successful for HBV genotypes C and E (from clinical samples) and D (plasmid sequence). Although we have not yet applied the method to other genotypes, we believe our methods are likely to be agnostic to genotype, as the primers were designed to be complementary to highly conserved regions of the HBV genome^15^. Sequencing of a mixed genotype-C/E sample demonstrates that the RCA approach is capable of identifying >1 genotype within a single sample without suggesting or introducing recombination events, illustrating the reliability of Nanopore long-read data for complete haplotype reconstruction. Further optimisation in sensitivity will be required before we can use the method to detect mixed infections in which one genotype is introduced as a minor variant. The methods developed in this study could potentially be applied to study other viruses with small, circular DNA genomes.

## Methods

### Patients and ethics

We used plasma samples from adults (aged ≥18 years) with chronic HBV infection attending outpatient clinics at Oxford University Hospitals NHS Foundation Trust, a large tertiary referral teaching hospital in the South-East of England. All participants provided signed informed consent for participation. Ethics permission was given by NHS Health Research Authority (Ref. 09/H0604/20). All methods and analysis were performed in accordance with the guidelines and regulations stipulated as part of the ethics approval. HBV DNA viral loads were obtained from the clinical microbiology laboratory (COBAS AmpliPrep/COBAS TaqMan, Roche^25^; a standard automated platform for quantification of viral loads). We chose samples for sequencing based on their high viral load; all were HBeAg-positive. Blood samples were collected in EDTA. To separate plasma, we centrifuged whole blood at 1800 rpm for 10 minutes. We removed the supernatant and stored in aliquots of 0.5–2 ml at −80 °C. We selected samples of minimum volume 0.5 ml and with a minimum HBV DNA viral load of 10^7^ IU/ml to optimize successful amplification and sequencing (Table 1).

### HBV plasmid

In addition to sequencing autologous HBV from clinical samples, we also applied our sequencing methods to a plasmid, in order to investigate the performance of our approach using a template for which the full molecular sequence is already known, and in which diversity is anticipated to be minimal or absent. We used the HBV 1.3-mer P-null replicon plasmid, a 6820 bp fully dsDNA construct, with a replication-deficient 1.3 × HBV length clone encoded along with ampicillin resistance genes and promoter sequences^17^. The plasmid was supplied as purified DNA in nuclease-free water.

### Nucleic acid extraction

For patient samples, we extracted total nucleic acid from 500 µl plasma using the NucliSENS magnetic extraction system (bioMérieux) and eluted into 35 µl of kit buffer as per the manufacturer’s instructions.

### Completion/ligation and Phi 29 rolling circle amplification

For patient samples, we prepared CL reactions in triplicate using previously described methods^16^. We modified this protocol to maximise the amount of DNA added, by using 6.4 μl extracted DNA plus 3.6 μl reaction mix to obtain a total reaction volume of 10 μl. We retained one reaction for sequencing after undergoing only the CL step, and the other two underwent RCA, using the previously described Phi 29 protocol^16^. The completion-ligation step was not required for the plasmid, so it directly underwent RCA using the same primers and laboratory protocol that were used for patient samples^16^. Primer sites are shown in Suppl Fig. 6.

### Library preparation and sequencing

For each sample, we used both the product of the CL reaction and the RCA reaction for library preparation using the Nextera DNA Library Preparation Kit (Illumina) with a modified protocol to account for lower input, based on a previously published method^26^. We sequenced indexed libraries, consisting of short fragments of PCR-amplified template, on a MiSeq (Illumina) instrument with v3 chemistry for a read length up to 300 bp paired-end.

We used the remaining RCA reaction products, consisting of concatemers of the unfragmented template DNA, for Nanopore sequencing. First, we resolved potential branching generated by RCA by digesting with a T7 endonuclease I (New England Biolabs). We carried out library preparation with a 1D Genomic DNA ligation protocol (SQK-LSK108, Oxford Nanopore Technologies, ONT), and sequenced the samples using R9.4 or R9.5.1 flowcells on a MinION Mk 1B sequencer (ONT).

### Analysis of Illumina data

We demultiplexed paired-end Illumina reads and trimmed low quality bases and adapter sequences (QUASR^27^ and Cutadapt^28^ software), before removing human reads by mapping to the human reference genome, hg19 using bowtie2^29^. We then used BWA-MEM^30^ to map non-human reads to HBV genotype A-H majority consensus sequences, derived from 4,500 whole genomes stored on HBVdb^31^. We used conventional numbering systems for the HBV genome, starting at the EcoR1 restriction site (G/AATTC, where the first T is nucleotide 1). We re-mapped the same reads using BWA-MEM to each within-sample majority consensus. In a test of accuracy, consensus genomes were locally aligned to contiguous elements (contigs) assembled ‘de novo’ from the trimmed reads (VICUNA software) and found to match perfectly.

### Analysis of nanopore sequence data: initial processing

We basecalled raw Nanopore reads of the RCA concatemers using ONT’s Albacore versions 2.0.2 (samples 1331 and 1332) and 2.1.10 (sample 1348 and 1331/1332 mix). We trimmed ‘pass’ reads (those with qscore >7) using Porechop v.0.2.3 (https://github.com/rrwick/Porechop) to remove adapter sequences. We used Kraken to classify reads^32^ against a custom database comprised of the human genome and all complete microbial genomes from RefSeq. We additionally mapped reads to a panel of reference sequences representing genotypes A-H (sequences available at https://github.com/hr283), in order to identify the genotype of the sample. These reference sequences had a repeat of the first 120 bp appended on the end, to ease the alignment of reads from circular genomes.

### Analysis of plasmid sequence

For the plasmid, raw Nanopore data was basecalled with guppy 1.8.10 and then trimmed with Porechop as previously. We constructed a custom reference sequence for use in the following alignment steps (sequence available at https://github.com/hr283). This had the same structure as the plasmid construct but used the sequence of the genotype D reference in the HBV sections. We removed a site from the reference which was known to be deleted in the plasmid, since our methods are not designed to call insertions and deletions with respect to the genotype reference (see further details below).

### Analysis of nanopore sequence data: error correction

Our initial consensus error correction procedure was adapted from the method previously described by Li *et al*.^12^. We started with complete concatemer sequences and chopped these into full or partial HBV genome reads (as illustrated in Fig. 1C). For this step, we identified repeat HBV genome reads in concatemeric sequences with the use of an anchor sequence comprising the first 100 bp of the relevant genotype reference. Reads were chopped every time the anchor sequence was found. Where individual anchor sequences were missed because of poor-quality data, we used the distance to the nearest anchor sequence as a guide to form individual genomes. Each HBV genome read was remapped with BWA-MEM^30^ to the HBV genotype reference. Note that since minimap2^33^ has recently replaced BWA-MEM for alignment of Nanopore data, future work would benefit from using minimap2 at the relevant steps in the pipeline.

Reads were assigned to either forward or reverse read sets, based on whether they mapped to the plus or minus strand of the genotype reference (Fig. 4(ii)). Concatemers containing reads in both sets were removed (representing a total of 13/1048 concatemers across all three patient samples). To select concatemers with *n* full genome reads for further analysis, we filtered for those containing ≥(*n* + 2) read-sections, since the first and last section of each concatemer are not guaranteed to be full length.

We applied our refined error correction method to complete concatemer sequences with ≥3 full genome reads (Fig. 4(i)). To speed up the search for true genetic variants, we only considered sites where a non-consensus base appeared at >60% frequency within one or more concatemers. We scored and filtered each of these potential variant sites using the following approach:

1. We conducted a Fisher’s exact test (https://pypi.org/project/FisherExact) to determine significance of the association between base and concatemer on forward and then reverse read sets (Fig. 4(iv)). If either of the resulting p-values were >0.01, we removed the site from the list of variants. We used the two p-values, p1 and p2, to generate a phred-based QUAL score by setting QUAL = −10 * log10(p1*p2), as reported in Suppl Table 3.
2. We calculated a strand bias p-value, by applying a chi squared contingency test to the numbers of forward vs reverse strand concatemers with vs. without observations of the variant base (defined as the most common non-consensus base). If this p-value was <0.01 then the potential variant was filtered out (Fig. 4(v)).

Sites failing either the concatemer-association or strand bias criteria were considered Nanopore errors, and were corrected using the consensus base across all concatemers. Note that to avoid false correction, if the most common base in the forward read set did not match the most common base in the reverse read set, then we defined the whole sample consensus base as ‘N’ (undetermined). Variant sites were corrected using the consensus base within each concatemer (Fig. 4(vi)). We additionally recorded the allele frequency, calculated as the proportion of base calls across all corrected concatemers that are equal to the most common non-consensus base. Further filtering based on allele frequency >10% was applied for consistency when comparing Nanopore variant calls with variants at >10% frequency in Illumina. These variants are shown in Suppl Table 3.

Whole-sample consensus Nanopore sequences were derived by taking the most common base at each site, if it was at >40% frequency and was the most common base in both the forward and reverse read sets, or calling the site as an ‘N’ otherwise. Note that the method is not designed to call insertions or deletions relative to the genotype reference; sites are only called as a gap (-) if there are no bases covering the site in either the forward or reverse read sets. The code used for data processing, error correction and variant calling is available on github: https://github.com/hr283/RCAcorrect.

### Sanger sequencing

Sanger sequencing was performed on the patient samples, using a pan-genotypic approach to generate multiple overlapping amplicons spanning the HBV genome (Suppl methods 2). The amplicons generated were examined for evidence of polymorphisms identified in both the Nanopore and Illumina sequencing data (Suppl Table 3, Suppl Figs 7 and 8).

### Phylogenetic trees

We generated maximum likelihood phylogenetic trees using RaxML^34^ with a gamma model of rate heterogeneity and a general time-reversible (GTR) nucleotide substitution model, followed by visualisation in FigTree.

## Supplementary information

Supplementary Data File
