# Supplementary material for: Illumina and Nanopore methods for whole genome sequencing of hepatitis B virus (HBV)
Source: Sci Rep. 2019 May 8;9:7081. doi: 10.1038/s41598-019-43524-9 (PMC6506499; doi:10.1038/s41598-019-43524-9)
Supplement: Supplementary file 1 — Supplementary Data File [file 41598_2019_43524_MOESM1_ESM.docx]

SUPPLEMENTARY DATA FILE

**Illumina and Nanopore methods for**

**whole genome sequencing of hepatitis B virus (HBV)**

Anna L McNaughton, Hannah E Roberts, David Bonsall, Mariateresa de Cesare,

Jolynne Mokaya, Sheila F Lumley, Tanya Golubchik, Paolo Piazza,

Jacqueline B Martin, Catherine de Lara, Anthony Brown, M Azim Ansari,

Rory Bowden, Eleanor Barnes, Philippa C Matthews

_______________________________________________________________

**CONTENTS**

- **Suppl Methods 1:** HBV-specific qPCR (page 3)
- **Suppl Methods 2:** Sanger sequencing (page 3)
- **Suppl Methods 3:** Quality scores for haplotype calls (page 3)
- **Suppl Table 1:** Relationship between number of genomes in HBV concatemer, number of Nanopore reads with at least that number of genomes, and average error rate of the Nanopore reads after correction (page 5).
- **Suppl Table 2:** Primers used for Sanger sequencing of full length HBV genome (page 6).
- **Suppl Table 3:** A list of sites at which genetic variants were identified in either Illumina or Nanopore HBV reads (page 7).
- **Suppl Fig 1:** The density of insert ends along the genome in CL and CL + RCA Illumina sequencing data from sample 1348 (page 8).
- **Suppl Fig 2:** Krona plots to illustrate the proportion of human vs virus (majority hepatitis B virus) reads in Illumina data derived from all samples (page 9).
- **Suppl Fig 3:** Illustration of Nanopore sequence data derived from a mixture of two plasma samples from adults with chronic HBV infection (ID 1331 and 1332, genotypes C and E, respectively), showing classification of 4799 single genome segments as either genotype C and E, and detailed visualisation of 6 unclassified segments (pages 10-11).
- **Suppl Fig 4:** Evidence of kmer-specific errors in Nanopore reads (page 12-13).
- **Suppl Fig 5:** Examples of kmer-specific errors in Nanopore HBV reads (page 14).
- **Suppl Fig 6:** Primer sequence locations for Sanger sequencing and rolling circle amplification (RCA) mapped onto the HBV genome (page 15)
- **Suppl Fig 7:** Errors in Sanger sequences relative to sites of sequencing primers, based on consensus generated by Illumina sequences (page 16)
- **Suppl Fig 8:** Sites of diversity in Sanger sequence chromatograms from samples 1331 and 1348 (page 17)
- **Suppl Fig 9:** Quality scores for haplotype calls (page 18)
- **References:** (page 19)

**Suppl Methods 1: HBV-specific qPCR**

We used HBV-specific qPCR to measure the yield of HBV DNA after rolling circle amplification (RCA) and compared with the non-amplified extracted DNA sample. The qPCR was adapted from Garson *et al*. [[3]](https://paperpile.com/c/3l13fx/FOpvU), using a SYBR Green approach to target a 98 bp region of the HBV surface (S) gene. We set up reactions using 2 μl sample DNA and 10 μl Power SYBR Green (Thermo Fisher) and performed thermal cycling using a LightCycler® 480 System, with the following conditions: 1 cycle at 95°C for 10 minutes, and 45 cycles at 95°C for 15 seconds and 60°C for 60 seconds. Prior to qPCR, RCA samples were diluted 100-fold to reduce the background DNA levels for SYBR Green qPCR, which were then compared with non-amplified extracted DNA from the same sample.

**Suppl Methods 2: Sanger sequencing**

We used a pan-genotypic Sanger sequencing approach adapted from Chook *et al* [[1]](https://paperpile.com/c/3l13fx/MZpCL) (Suppl Table 2), in which the HBV genome is sequenced as seven overlapping fragments by nested PCR. We added an additional fragment (derived using primer sets E and 7) to the original protocol to improve coverage of the long region amplified using primers 595f and 1797r. Primer sites are shown in Suppl Fig 3 and PCR reactions were performed as previously described [[1]](https://paperpile.com/c/3l13fx/MZpCL).

We stained amplified products to verify by agarose gel electrophoresis under UV light, and sequenced using an ABI 3730 / 3730xl capillary sequencer (Department of Zoology, University of Oxford). We aligned fragments using SSE software [[2]](https://paperpile.com/c/3l13fx/K81w) and derived a consensus sequence. Errors in Sanger sequences (defined as differences from Illumina-derived consensus) largely corresponded to sites of sequencing primers (Suppl Fig 7).

**Suppl Methods 3: Quality scores for haplotype calls**

We designed a possible method for assigning quality scores to haplotype calls as follows:

(1) Empirically estimate the probability of a mismatch being called at any one site when taking the consensus sequence of a concatamer with n full genome reads, for values of n from 3 upwards. The values of n that are of interest will depend on the size of the dataset). This can be done by selecting all concatemers with exactly n full genome reads (+2 partial genome reads), generating a consensus sequence for each concatemer and calculating the average mismatch rate compared to the whole sample consensus, across all sites at which there is a very low probability of variation. In our case, we used the sites with <1% variation in Illumina data as the set of sites with a very low probability of variation

(2) Estimate the probability of miscalling a haplotype from a concatemer containing **n full genomes**. Let the number of sites under consideration, i.e the number of sites identified as polymorphic, be v. The probability of miscalling a haplotype is equal to:

1 - Prob(all sites called correctly)

= 1 - (1-Prob(miscall at a site))^v^

Where Prob(miscall at a site) is the value estimated in (1) above.

(3) Let p(n) be the probability calculated in (2) above. A phred-based qual score can be calculated for a haplotype based on the number of observations of that haplotype and the length of the concatemers involved. For example, taking a simple case where we have **m observations of a haplotype**, all resulting from concatemers containing 5 full genomes, the qual score would be -10log10(p(5)^m^). A contour plot showing how this qual score varies for different values of n and m is shown in Suppl Fig 9.

**Suppl Table 1: Relationship between number of genomes in HBV concatemer, number of Nanopore reads with at least that number of genomes, and average error rate of the Nanopore reads after correction.** Data based on sample from an adult with chronic HBV infection with viral load >10^8 IU/ml, recruited in Oxford (sample ID 1331). To correct the reads, the consensus within each concatemer has been used.

| **Number of complete genomes (g) in concatemer** | **3** | **4** | **6** | **8** |
| --- | --- | --- | --- | --- |
| **Number of reads with ≥ g complete genomes** | 208 | 158 | 84 | 41 |
| **Average error rate after consensus correction (%)** | 0.88 | 0.73 | 0.57 | 0.51 |

**Suppl Table 2: Primers used for Sanger sequencing of full length HBV genome.** Primers adapted from Chook *et al*.[[1]](https://paperpile.com/c/3l13fx/MZpCL) The primers used for sequencing each amplicon are indicated (*). Primer 1797 (**) is used twice in the first round reactions, as a part of set A and set E.

|  | **Outer primers** | | | **Inner primers** | | | |
| --- | --- | --- | --- | --- | --- | --- | --- |
| **Set** | **Primer** | **Sequence** | **Tm (°C)** | **Set** | **Primer** | **Sequence** | **Tm (°C)** |
| **A** | 251F | GACTYGTGGTGGACTTCTC | 54 | **1** | 251F* | GACTYGTGGTGGACTTCTC | 54 |
|  |  |  |  |  | 1190R | TCAGCAAAYACTYGGCA |  |
|  | 1797R** | CCAATTTMTGCYTACAGCCTC |  | **2** | 595F* | CACHTGTATTCCCATCCCA | 54 |
|  |  |  |  |  | 1797R | CCAATTTMTGCYTACAGCCTC |  |
| **B** | 2300F | CCACMWAATGCCCCTATC | 50 | **3** | 2807F* | CGCHTCATTTTGYGGGTCAC | 52 |
|  |  |  |  |  | 617R | GAYGAYGGGATGGGAATACA |  |
|  | 654R | GSCCCAMBCCCATAGG |  | **4** | 2300F* | CCACMWAATGCCCCTATC | 50 |
|  |  |  |  |  | 215R | AGRAAMACMCCGCCTGT |  |
| **C** | 1859F | ACTNTTCAAGCCTCCRAGCTG | 52 | **5** | 1877F | CTGTGCCTTGGRTGGCTT | 52 |
|  | 2835R | GTTCCCAVGWATAWGGTGAYCC |  |  | 2835R* | GTTCCCAVGWATAWGGTGAYCC |  |
| **D** | 1584F | ACTTCGMBTCACCTCTGCACGT | 57 | **6** | 1584F | ACTTCGMBTCACCTCTGCACGT | 57 |
|  | 2396R | GTCKGCGAGGYGAGGGAGTT |  |  | 2331R* | GAAGYGTKGAYARGATAGGGGCATT |  |
| **E** | 1190F | AYGCAACCCCCACTGG | 51 | **7** | 1190F* | AYGCAACCCCCACTGG | 51 |
|  | 1797R** | CCAATTTMTGCYTACAGCCTC |  |  | 1797R | CCAATTTMTGCYTACAGCCTC |  |

**Suppl Table 3: A list of sites at which genetic variants were identified in either Illumina or Nanopore HBV reads.** Sites are included that have either (a) >10% variation in Illumina RCA rep1, or (b) variant allele frequency > 10%, both concatemer-association p-values < 0.01, and strand bias p-value > 0.01 in the Nanopore data. The QUAL score is based on a combination of the two concatemer-association p-values (see methods). QUAL scores are capped at 66 as this corresponds to the minimum p-values reported by the Fisher’s Exact test.

| **Sample ID** | **Genome position** | **Nanopore QUAL score** | **Variant frequency in Illumina RCA rep 1 (%)** | **Variant allele frequency in Nanopore (%)** |
| --- | --- | --- | --- | --- |
| 1331 | 1041 | 66 | 17 | 18 |
| 1331 | 1054 | 66 | 20 | 19 |
| 1331 | 1936 | 66 | 39 | 29 |
| 1331 | 2134 | 66 | 25 | 27 |
| 1348 | 400 | 66 | 5 | 11 |
| 1348 | 841 | 66 | 8 | 11 |
| 1348 | 915 | 66 | 11 | 14 |
| 1348 | 1425 | 66 | 21 | 19 |
| 1348 | 2189 | 66 | 5 | 12 |

**Suppl Fig 1: The density of insert ends along the genome in CL and CL + RCA Illumina sequencing data from HBV sample 1348.**

**
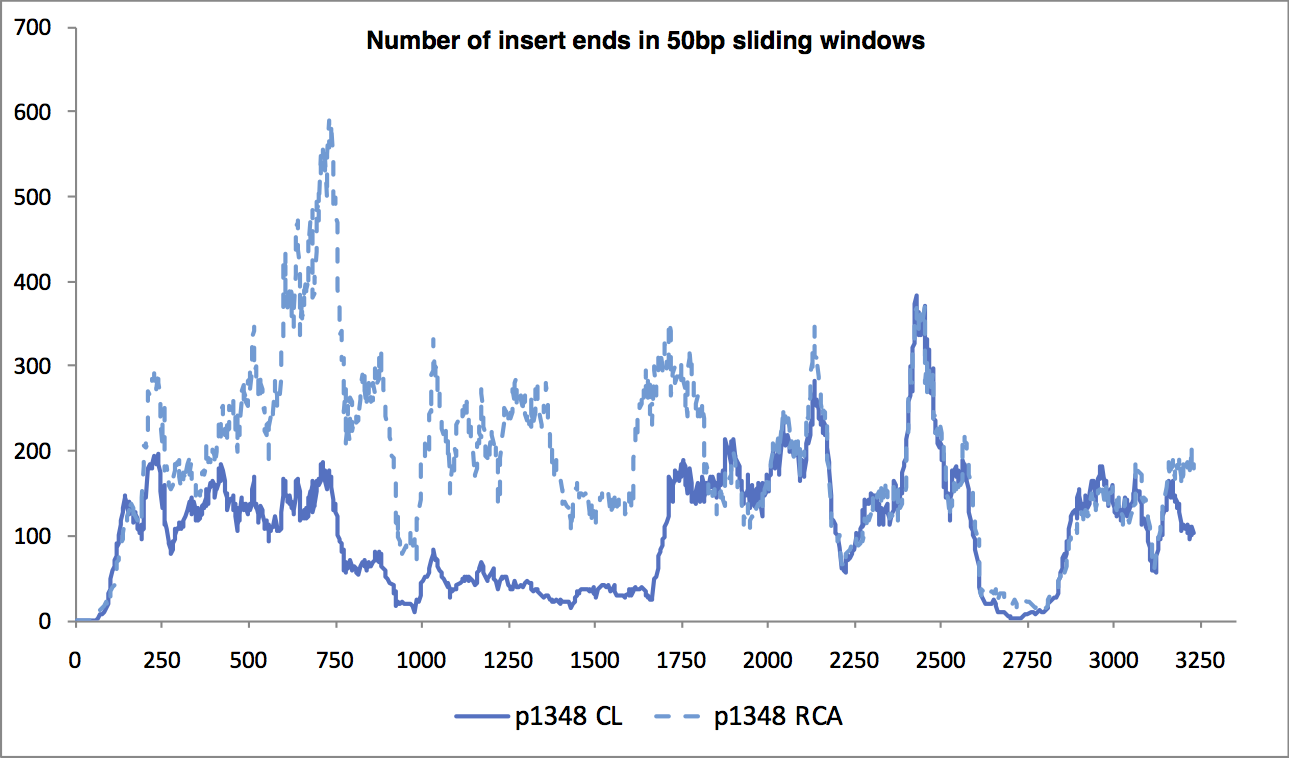
**

**Suppl Fig 2: Krona plots to illustrate the proportion of human vs virus (majority hepatitis B virus) reads in Illumina data derived from three plasma samples.**

A: Sample 1331. B: Sample 1332. C: Sample 1348

**
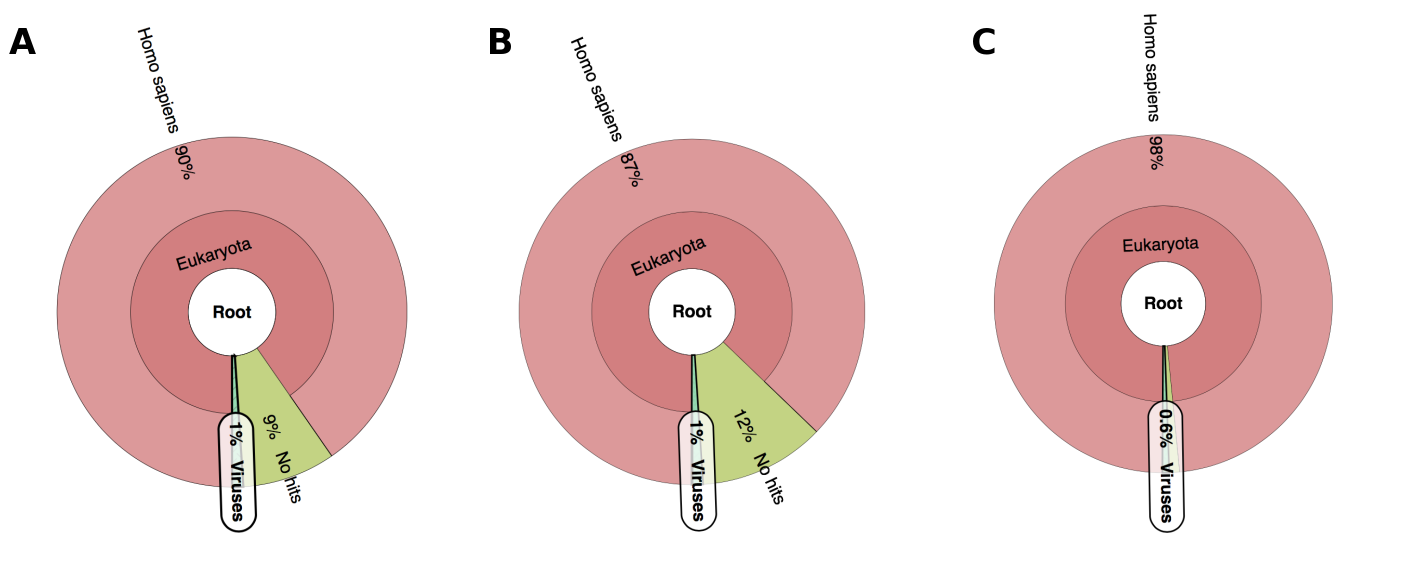
**

**Suppl Fig 3: Illustration of Nanopore sequence data derived from a mixture of two plasma samples from adults with chronic HBV infection (ID 1331 and 1332, genotypes C and E, respectively), showing classification of 4799 single genome segments as either genotype C and E, and detailed visualisation of 6 unclassified segments**.

A: Plot showing the similarity of read segments to genotype C vs E, as assessed by the aligned bases at 335 genotype-discordant sites. Each point represents one of 4805 genome segments contained within concatemeric reads derived from the mixed sample. These segments have been mapped, using BWA-MEM [[4]](https://paperpile.com/c/3l13fx/6iXU), to a fasta file containing both a genotype C and genotype E reference sequence, and points are coloured according to which genotype the majority of segments within the concatemer mapped to (red = C, yellow = E). Points are ordered along the x-axis according to read name. The vast majority (4799) segments can be classified as either genotype C (proportion C > 0.8) or E (proportion C < 0.2). No segment has a classification that differs from the majority genotype of the concatemer (as represented by the colour).

B: A representation of the 6 concatemers containing segments that could not be classified as either genotype C or E. Coloured columns represents genotype-discordant sites, arranged in the order they appear along the genome. For each segment these are coloured as red (match to C/1331 consensus), yellow (match to E/1332 consensus) or grey (match to neither/missing data). Columns on the left hand side indicate the read name, segment start, majority genotype from BWA-MEM mapping, and proportion genotype C, where cells containing a proportion between 0.2 and 0.8 are highlighted in green. 4/6 of these segments cover less than 8 genotype-discordant sites, the remaining two appear to show the characteristics of poor quality sequence data, matching to a mixture of gt C, gt E, or neither along the whole length of the segment.

Fig 3A:


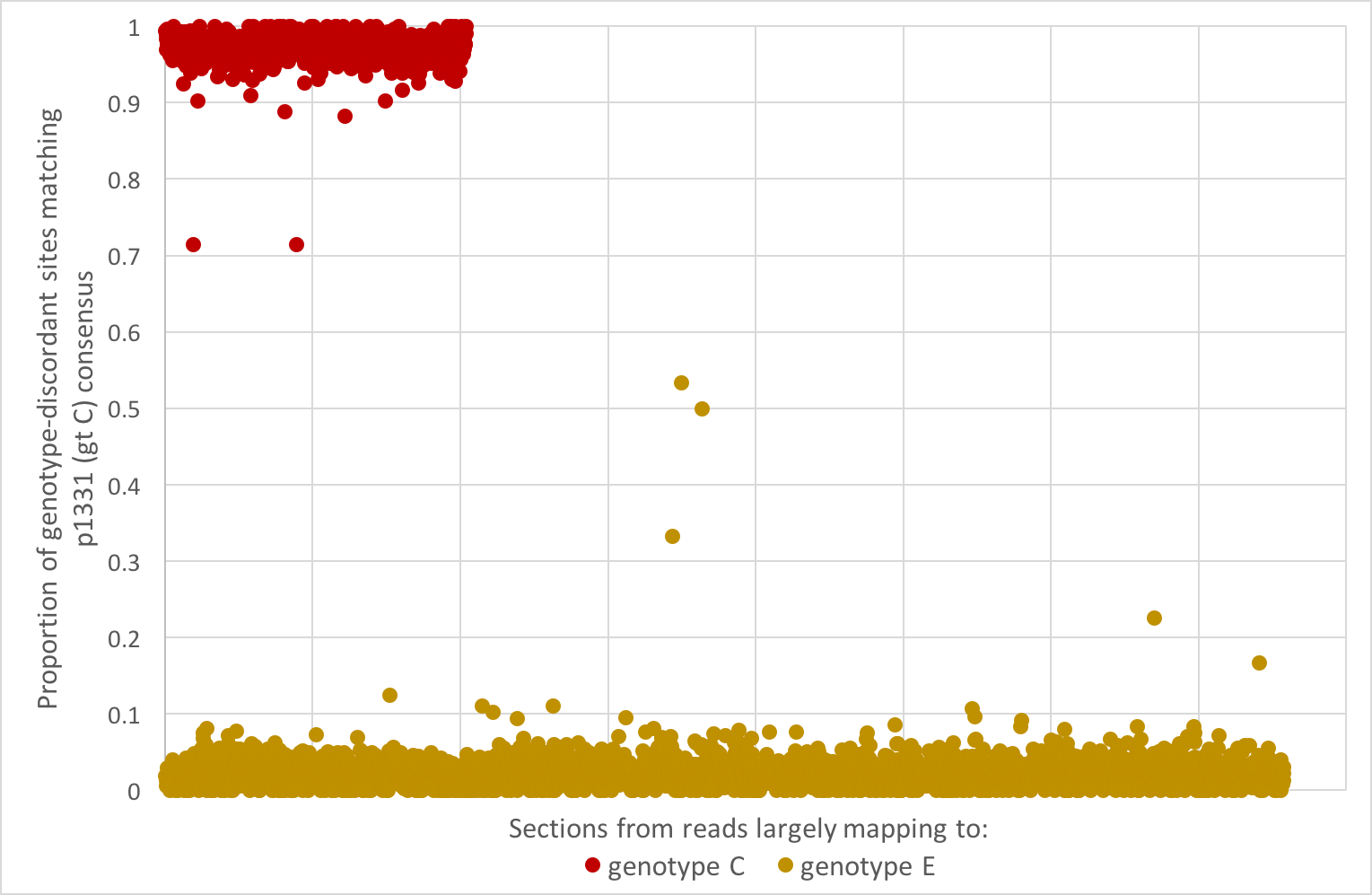


Fig 3B:


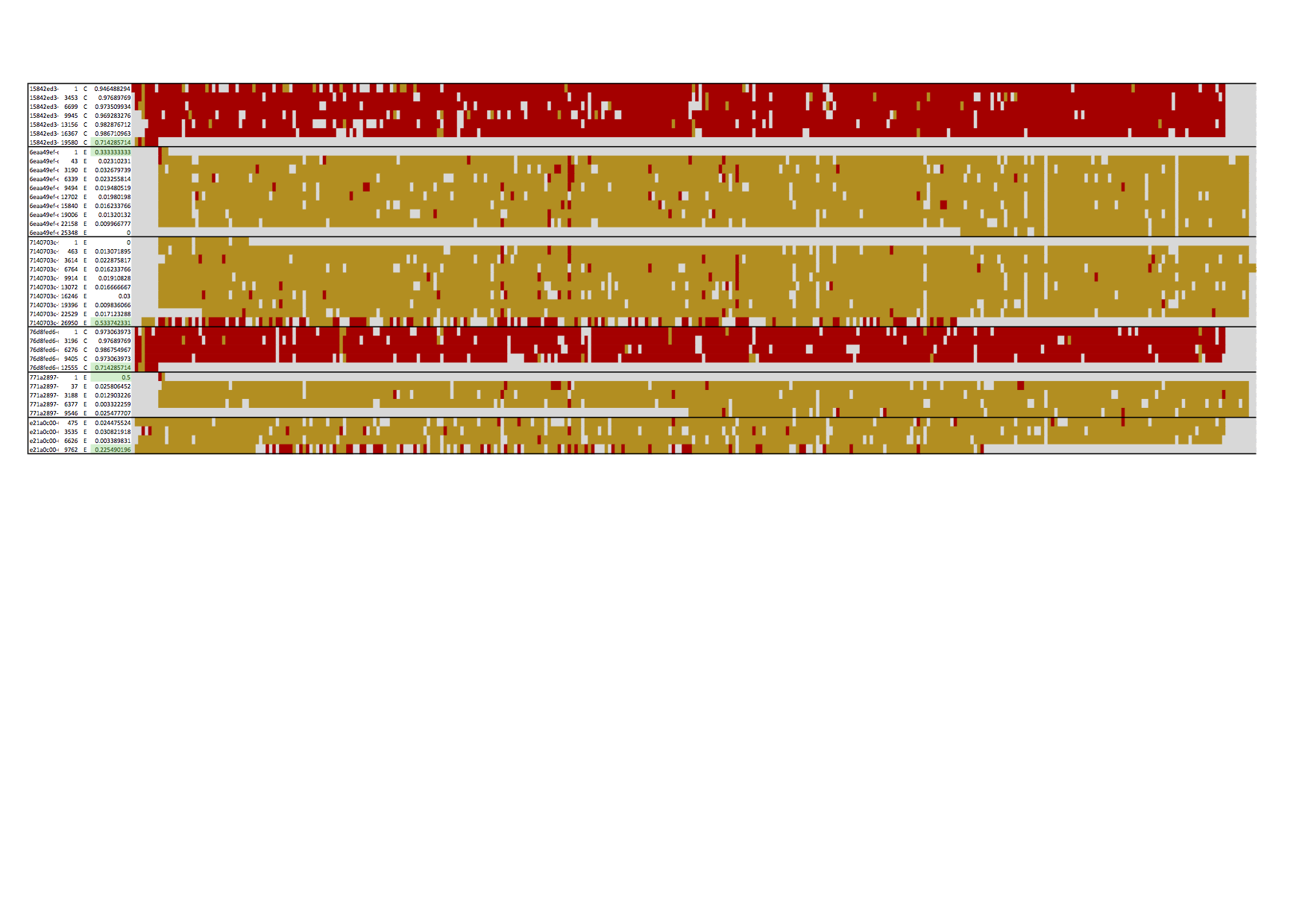


**Suppl Fig 4. Evidence of kmer-specific errors in Nanopore reads.** (A) Error rate for all 5-mers, ordered alphabetically along the x-axis, shown in blue. The error rate for the corresponding reverse complement 5-mer is shown in orange. (B) Error rate for all 5-mers beginning ‘AAC’, with colours as in (A), showing differences in error rate between kmers and their reverse complements. The error rates are calculated based on a batch of 10000 Nanopore reads of the human reference cell line NA12878, basecalled with Albacore v 2.0.2 (Oxford Nanopore Technologies) and mapped to the GRCh37 build of the human genome. This dataset comprised ~67Mb of aligned bases. For each kmer, the number of times it had been called with an error at the 4th position (or for insertions, in between positions 3 and 4) compared to the reference sequence was counted. The reference sequence was assumed to be the true kmer sequence. To get the error rate, this number was divided by the total number of times the kmer had been read (either correctly or incorrectly). This process was then repeated, but counting mismatches/deletions at the 2nd position and insertions between the 2nd and 3rd position. This gave corresponding error rates for reverse complement kmers. For example, with reference to (B), the kmer AACGC, when read in the forward direction, contains a mismatch/deletion at the G or insertion before the G 38% of the time. When read in the reverse complement orientation, GCGTT, a mismatch/deletion at the C or insertion after the C is observed 11% of the time. Both of these categories of errors, when aligned to the reference sequence, would be observed as an error at or just after the G, but these errors would be much more frequent in forward strand reads than reverse strand reads. The exact error profile may change for different flow-cell chemistries and basecallers, but the principle of strand-biased errors being observed as a result of fluctuations in the kmer error profile remains the same.

**A:
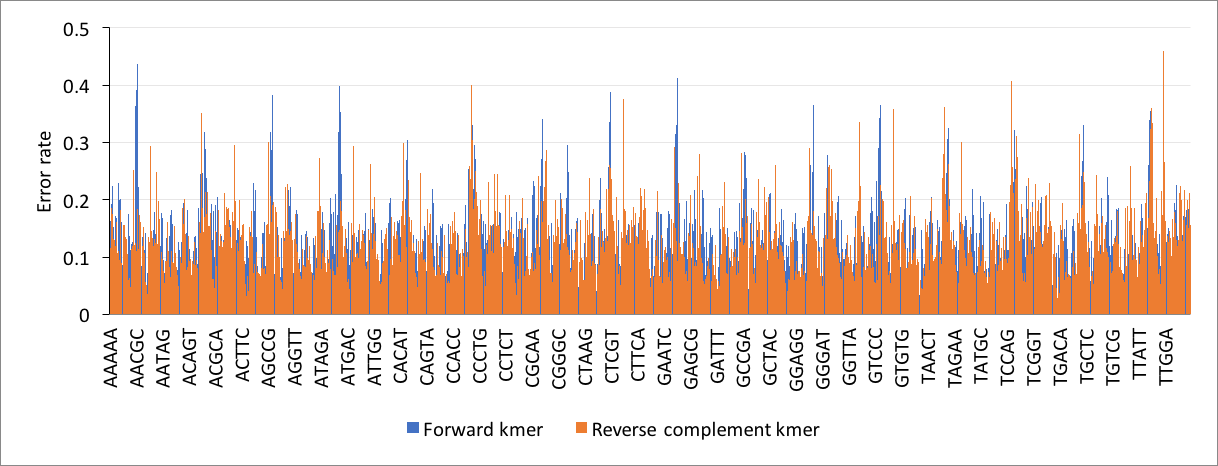
**

**B:**

**
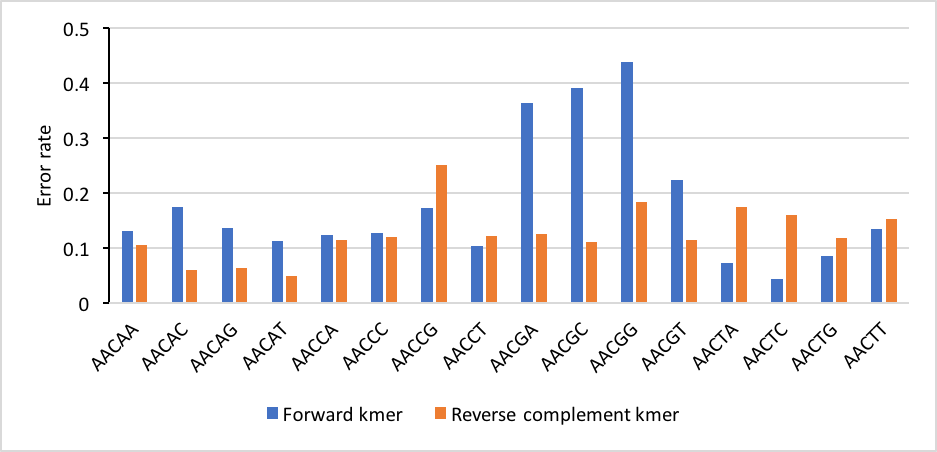
**

**Suppl Fig 5. Examples of kmer-specific errors in Nanopore HBV reads.** Snapshots from the Integrative Genomics Viewer (IGV)[[5]](https://paperpile.com/c/3l13fx/9NPG) for sample 1331 are shown. Reads have been aligned to the genotype C reference, the sequence of which is shown at the top of each panel. Coverage is indicated by the grey columns below the reference sequence. For sites with >10% variation, these columns are coloured according the the distribution of bases at that site. Read-sections are grouped according to the concatemers they originated from and coloured by strand (+ = red, - = blue). Insertions, deletions and mismatches are indicated as described in Suppl Fig 1. The three snapshots are centred on positions 1150, 1384 and 1914 (from L to R). In each of the cases shown the variant alleles occur almost exclusively in reads from only one of the strands (at sequence positions 1149, 1151 and 1914 the variant appears in the - strand, while in 1383 and 1915 the variant is only seen in the + strand).

**
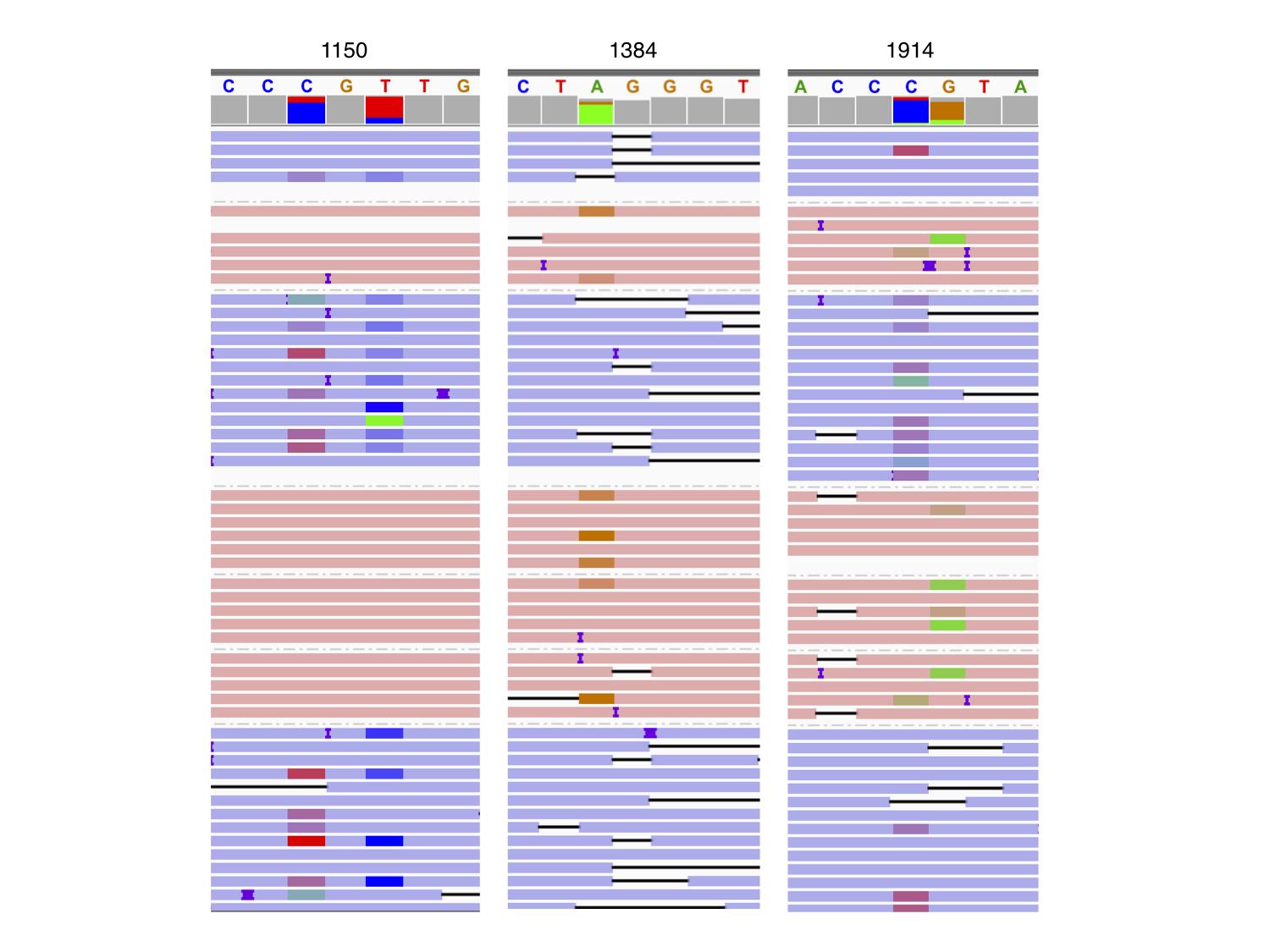
**

**Suppl Fig 6. Primer sequence locations for Sanger sequencing and rolling circle amplification (RCA) mapped onto the HBV genome.** Primer locations are indicated with arrows. For the Sanger sequencing primers, triangles have also been used to illustrate the locations of additional primers used for amplification of the inner fragments during the nested PCR (see Suppl Table 2 for more details). Grey boxes indicate a region of the genome, at approximately nt 2500-2700, that was found to have a consistent drop in coverage with all sequencing approaches. A plot of the average pairwise distance of HBV sequences, both within and between genotypes, is also shown to highlight diverse regions of the genome.


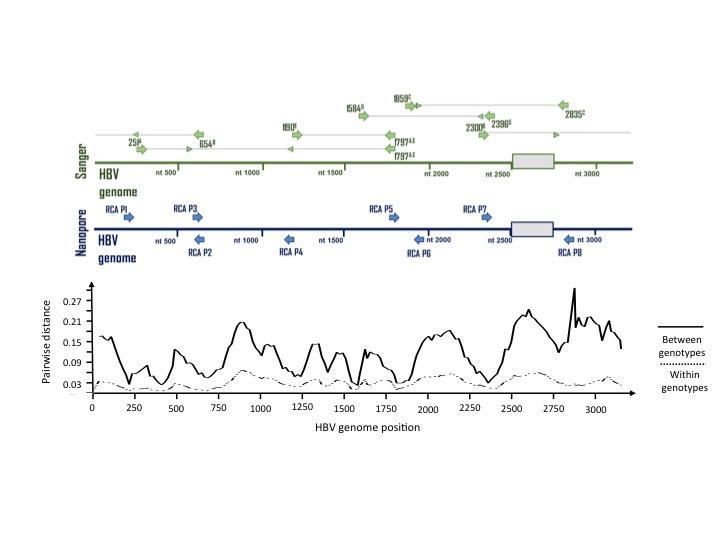


**Suppl Fig 7: Errors in Sanger sequences relative to sites of sequencing primers, based on consensus generated by Illumina sequences.** Errors shown for sequences derived from adults with chronic HBV infection with viral load >10^8 IU/ml, recruited in Oxford (sample ID 1331 and 1332). Derived Sanger sequences for 1331 and 1332 were 3215 bp and 3172 bp in length respectively, with the reduction in length of 1332 largely accounted for by a poorly sequenced region at nt 2843-2882 due to a short overlap between two of the overlapping fragments.


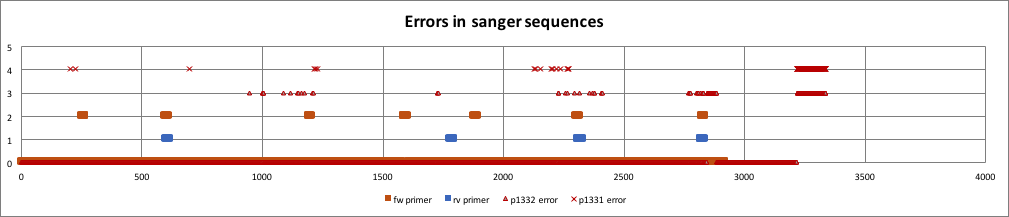


**Suppl Fig 8: Sites of diversity in Sanger sequence chromatograms from samples 1331 and 1348.** Sites of diversity were identified in Nanopore and Illumina sequencing data, as listed in Suppl Table 3 and searched for in Sanger sequences spanning the relevant sites. Sites at nucleotide position 400 in sample 1348 are not shown due to the poor sequencing quality at this site making it difficult to infer if diversity is present at the site. Each site has been highlighted in the region shown and the genome position is cited.


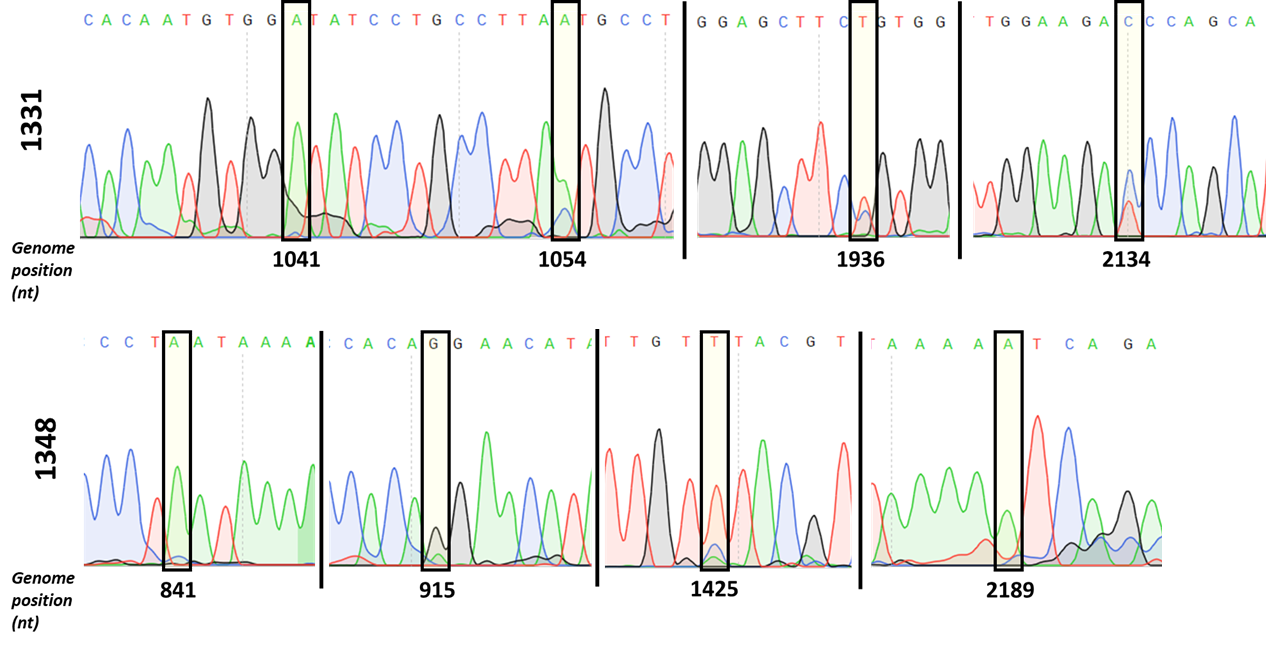


**Suppl Fig 9: Quality scores for haplotype calls.** Phred-based quality scores are calculated based upon the probability of a haplotype call being incorrect, as described in Suppl Methods 3. The scores shown here are from simple cases where all the concatemers supporting a haplotype contain the same number of full HBV genomes. The values in this heat map are calculated based on data from sample 1331.

**
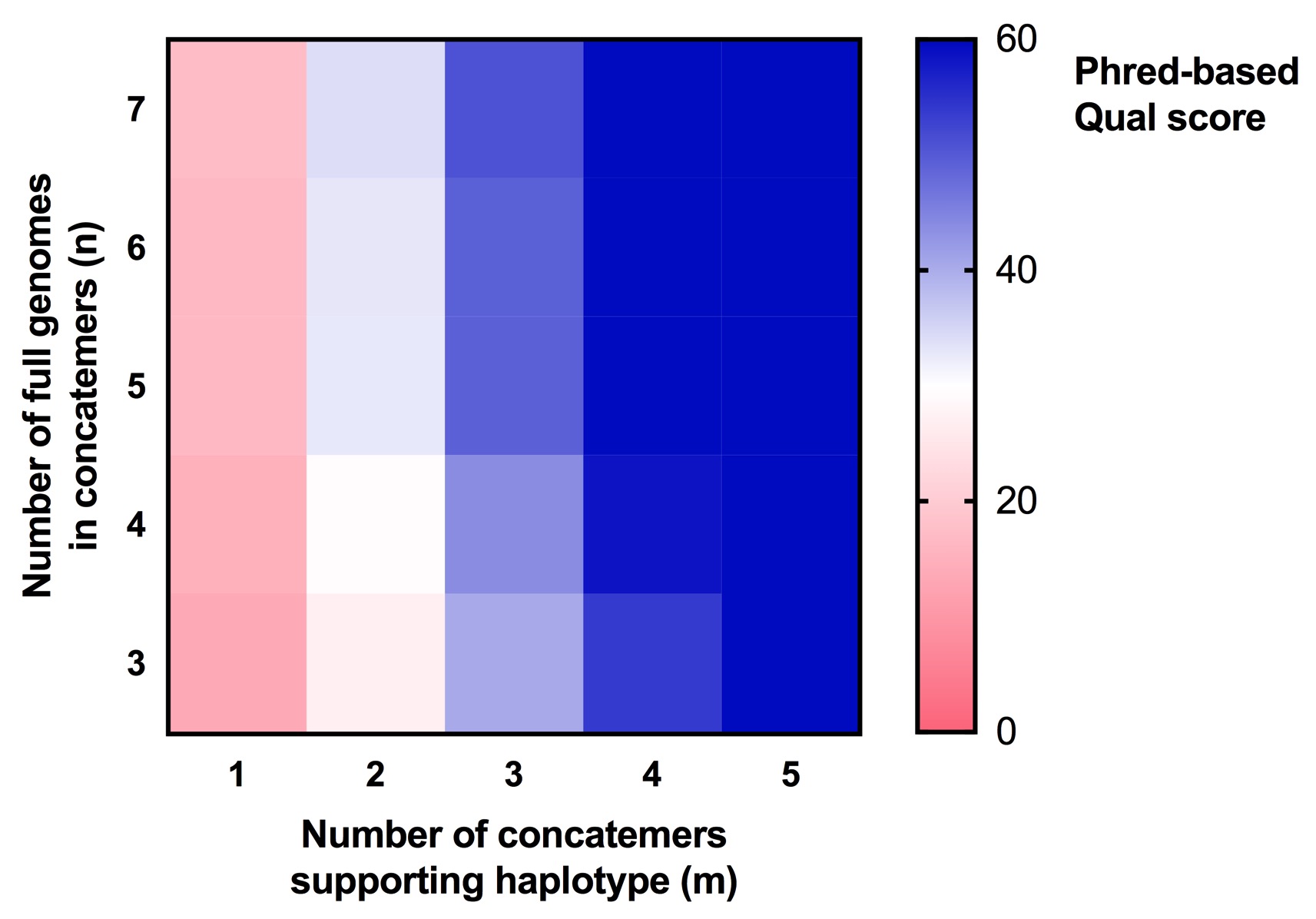
**

**References for supplementary material:**

1. [Chook JB, Teo WL, Ngeow YF, Tee KK, Ng KP, Mohamed R. Universal Primers for Detection and Sequencing of Hepatitis B Virus Genomes across Genotypes A to G. J Clin Microbiol. 2015;53: 1831–1835.](http://paperpile.com/b/3l13fx/MZpCL)

2. [Simmonds P. SSE: a nucleotide and amino acid sequence analysis platform. BMC Res Notes. 2012;5: 50.](http://paperpile.com/b/3l13fx/K81w)

3. [Garson JA, Grant PR, Ayliffe U, Ferns RB, Tedder RS. Real-time PCR quantitation of hepatitis B virus DNA using automated sample preparation and murine cytomegalovirus internal control. J Virol Methods. 2005;126: 207–213.](http://paperpile.com/b/3l13fx/FOpvU)

4. [Li H. Aligning sequence reads, clone sequences and assembly contigs with BWA-MEM. arXiv preprint. 2013; Available:](http://paperpile.com/b/3l13fx/6iXU) <http://arxiv.org/abs/1303.3997v2>

5. [Robinson JT, Thorvaldsdóttir H, Winckler W, Guttman M, Lander ES, Getz G, et al. Integrative genomics viewer. Nat Biotechnol. 2011;29: 24–26.](http://paperpile.com/b/3l13fx/9NPG)
